# Role of Hypoxia and Rac1 Inhibition in the Metastatic Cascade

**DOI:** 10.3390/cancers16101872

**Published:** 2024-05-14

**Authors:** Enikő Tátrai, Ivan Ranđelović, Sára Eszter Surguta, József Tóvári

**Affiliations:** 1 The National Tumor Biology Laboratory, Department of Experimental Pharmacology, National Institute of Oncology, H-1122 Budapest, Hungary; randelovic.ivan@oncol.hu (I.R.); surguta.sara@ext.oncol.hu (S.E.S.); tovari.jozsef@oncol.hu (J.T.); 2 School of Ph. D. Studies, Semmelweis University, H-1085 Budapest, Hungary

**Keywords:** hypoxia, hypoxia-inducible factors, metastasis, small G-proteins

## Abstract

**Simple Summary:**

In recent years, several studies have demonstrated the negative impact of tissue hypoxia on tumor pathology. In a significant number of cases, tumors develop resistance to chemo- and radiotherapy, fostering a more aggressive phenotype of tumor cells. The role of small regulatory proteins in cell motility is well known. In our previous work, we have investigated cell proliferation and motility, as well as expression patterns of factor which induce hypoxia and small regulatory proteins responsible for motility, in tumor cell lines of different tissue origins cultured under hypoxic conditions. In this review, we aim to highlight the significance of inhibiting motility factor mitigating metastasis under hypoxic conditions. We have discussed the effect of hypoxia on metastasis formation and the role of motility factor in this process. Although the molecular link between hypoxia-induced factor and small regulatory protein of motility remains unclear, findings primarily from in vitro studies so far suggests that targeting motility factor and its inhibition could be an effective complementary therapeutic strategy for suppressing tumor metastatic formation.

**Abstract:**

The hypoxic condition has a pivotal role in solid tumors and was shown to correlate with the poor outcome of anticancer treatments. Hypoxia contributes to tumor progression and leads to therapy resistance. Two forms of a hypoxic environment might have relevance in tumor mass formation: chronic and cyclic hypoxia. The main regulators of hypoxia are hypoxia-inducible factors, which regulate the cell survival, proliferation, motility, metabolism, pH, extracellular matrix function, inflammatory cells recruitment and angiogenesis. The metastatic process consists of different steps in which hypoxia-inducible factors can play an important role. Rac1, belonging to small G-proteins, is involved in the metastasis process as one of the key molecules of migration, especially in a hypoxic environment. The effect of hypoxia on the tumor phenotype and the signaling pathways which may interfere with tumor progression are already quite well known. Although the role of Rac1, one of the small G-proteins, in hypoxia remains unclear, predominantly, in vitro studies performed so far confirm that Rac1 inhibition may represent a viable direction for tumor therapy.

## 1. Introduction

An adequate supply of oxygen is essential for cell growth and proliferation, and it is influenced by the distance between cells and blood vessels. A distance exceeding 100 μm from the cell to the nearest vessel is considered critical [1]. The atmospheric oxygen level is 21% (160 mmHg), and approximately the same level is found in the case of cultured cells within a thermostat. Usually, this oxygen level is commonly referred to as normoxia, although it differs from actual peripheral tissue oxygenation in the living organisms. During breathing, the oxygen level in lung alveoli is around 14.5% (110 mmHg), while in peripheral tissues it decreases to 3.4–6.8% [2]. Metazoans utilize hypoxic signaling to adapt cells to oxygen deprivation [3]. The primary regulators in this adaptation are hypoxia-inducible factors (HIFs), which, as transcription factors, affect and regulate the expression of hundreds of genes involved in glucose metabolism, cell proliferation, migration and angiogenesis. Due to continuous and intense tumor growth, a hypoxic state develops quickly. Hypoxia plays a crucial role in the development of both metastasis and therapeutic resistance [4].

In our previous study, we performed experiments involving tumor cell lines with different tissue origins. Some of these cell lines exhibited heightened migratory and me-tastatic potential, with an intensive migration capability under hypoxic conditions [5]. The presence of hypoxia and the expression of hypoxia-inducible transcription factors have been associated with elevated rates of distant metastasis and poorer survival across many tumor types [6]. Moreover, hypoxia regulates tumor metastasis through various mechanisms including the epithelial–mesenchymal transition (EMT), intravasation, extravasation, homing and the premetastatic niche [6]. Interestingly, a preclinical study demonstrated that respiratory hyperoxia (60% O_2_) diminished intratumoral hypoxia, leading to tumors [7].

The importance of hypoxia is well known in solid tumors, where it is considered as an independent prognostic marker in head and neck cancer [8]. Additionally, the expression of HIF-1α serves as an important prognostic indicator in glioblastoma and renal cell carcinoma [9,10]. Tumor hypoxia can be classified as acute, chronic and cyclic hypoxia. (1) Acute hypoxia arises from a temporary blockage of capillaries caused by the pressure of the growing tumor mass or irregular erythrocyte flow, typically lasting for a shorter time (less than 6 h). (2) Chronic hypoxia occurs when tumor cells extend beyond 100 µm from the nearest blood vessels [11]. (3) Cyclic hypoxia involves the transient blockage of inefficient tumor vasculature resulting in periodic cycles of hypoxia and reoxygenation. The duration of hypoxia–reoxygenation cycles can last from minutes to days [12]. Under cyclic hypoxia, when the O_2_ supply is fluctuating, cancer cells have shown a greater metastatic capacity in mouse models of human cancers compared to chronic hypoxia [13]. Moreover, cyclic hypoxia may enhance the angiogenesis capacity [14] and promote stem-like properties in breast cancer and neuroblastoma cells [15], as well as result in the greater selection of gastric cancer cells with elevated self-renewal and survival properties compared to chronic hypoxia [16]. Studies have shown that cyclic hypoxia accelerates tumor growth through HIF-1α mediated adaptation [17]. While most in vitro experiments focus on chronic hypoxia, the emerging evidence suggests that cyclic hypoxia may play a greater role in the development of aggressive tumor phenotypes [18].

## 2. Role of HIFs in Hypoxia

Hypoxia-inducible factors (HIFs) regulate cellular adaptation to low oxygen levels by activating the expression of numerous genes, which encompass various functions such as cell immortalization, metabolic reprogramming, vascularization and the epithelial–mesenchymal transition (EMT) [19]. These genes include transcription factors, chromatin modifiers, enzymes, receptors, transporters, adhesion molecules, cell surface molecules, membrane proteins and microRNAs [20]. Among the HIF proteins, HIF-1 is the most well characterized.

HIF-1 consists of two subunits, alpha and beta, forming a heterodimeric protein. In normoxia, HIF-1α binds to the von Hippel–Lindau (VHL) protein, triggering the ubiquitin ligase system, which leads to the proteasomal degradation of HIF-1α through the post-translational hydroxylation of proline residues [21]. The oxygen-dependent degradation domain (ODDD) of the HIF-1α region has both N- and C-terminal parts, which can interact independently with VHL. This domain is responsible for the hydroxylation of two prolyl residues [22]. Additionally, there are two transactivation domains (TADs), the N-terminal and C-terminal, within the HIF-1α subunit. The hydroxylation reaction is mediated by the factor inhibitor HIF (FIH), which blocks the interaction between the C-TAD and CH-1 domain of the p300 transcriptional coactivator. Consequently, HIF-1 is not able to connect to the HREs under normoxic conditions [23]. There are other participants in the HIF-1 regulation such as the Constitutive photomorphogenesis mutant 9 (COP9) signalosome (CSN) holocomplex [24]. CSN5 interacts directly with both the CODD (C-terminal oxygen-dependant degradation domain) of HIF-1α as well as the pVHL E3 ligase, so pVHL and the PHDs interact with the same region of HIF-1α. Increased CSN5 expression is sufficient for aerobic HIF-1α stabilization, and its overexpression is associated with tumor progression. CSN5 mRNA expression was downregulated by prolonged and severe hypoxia [25]. Another participant of HIF regulation is the hypoxia-associated factor (HAF), an ubiquitin ligase specific for HIF-1α, leading to its ubiquitination and proteasomal degradation through an oxygen-independent way [25].

**Figure 1:**
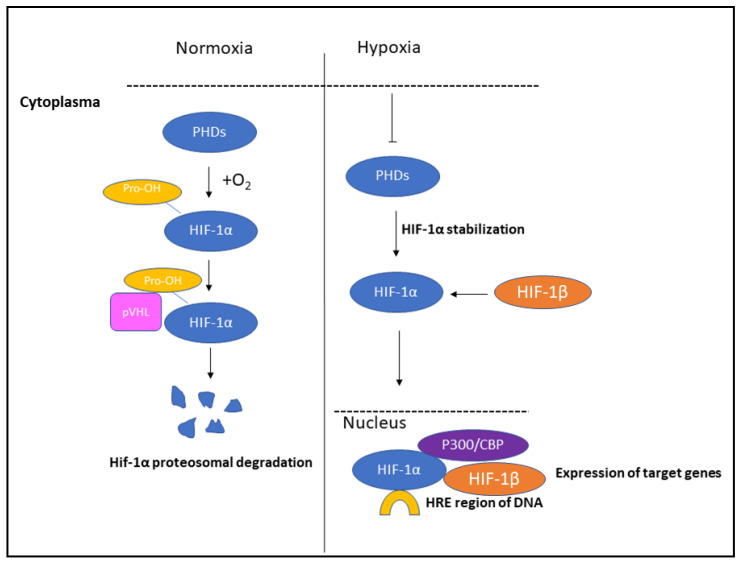
The role of HIF-1α under normoxic and hypoxic conditions. Under normoxia, HIF-1α is degraded by proteosomal degradation, while under hypoxic conditions, it binds to HIF-1β and translocates to the nucleus where it binds to the HRE region of DNA.

During hypoxia, prolyl-hydroxylases (PHDs) become inactive, resulting in the stabilization and dimerization of HIF-1α with HIF-1β (also called ARNT). This complex then translocates to the nucleus and binds to hypoxia response elements (HREs) (Figure 1). The consensus HRE sequence is 5′-(A/G)CGTG-3′ [19]. The parts of the helix loop helix (HLH) and Per ARNT Sim (PAS, a molecular sensor) domains (aa 1–166) are sufficient for heterodimerization, while the DNA binding of the HIF-1α/HIF-1β heterodimer requires the presence of HIF-1α amino acids 1–390 [26].

HIF-2α shares 48% amino acid sequence homology with HIF-1α and exhibits a very similar domain structure and regulation features in vitro, including induction within a few minutes by hypoxia [27]. Initially identified in endothelial cells, HIF-2α is also expressed in the parenchyma and interstitial cells of various organs [28]. HIF-2α is expressed mostly during embryonic development and in adult vascular endothelial cells in the lungs, placenta and heart [29]. In gastrointestinal cancers, it has been proven that HIF-2α has an impact on cellular proliferation, angiogenesis, apoptosis, metastasis and resistance to chemo- and radiotherapy [28].

HIF-3α is less well-known and studied due to its multiple variants [29]. While some variants act as dominant-negative regulators of HIF-1α and HIF-2α, others HIF-3α variants inhibit HIF-1α or HIF-2α by competing for the common HIF-β subunit [30]. It is expressed in the thymus, lung, brain, heart and kidney of adult mice, forming heterodimers with HIF-1β [31]. HIF-3α mRNA levels are tissue-specific in zebrafish, but not in mammals [32]. Another splice variant of mouse HIF-3α, known as the neonatal and embryonic PAS (Per ARNT Sim), is expressed during late embryonic and early postnatal stages. It dimerizes with HIF-1β and indirectly inhibits HIF-1 and HIF-2α activity [32].

## 3. Hypoxia-Mediated Tumor Progression and Metastatic Process

Metastasis is a complex process which consists of many different steps. Hypoxia can influence on metastasis at several points by activating the HIFs. Tumor cells undergo phenotypic changes in response to external stimuli, leading to alterations in their morphology, mirrored in EMT alterations and increased motility. Thus, these changes contribute to the heightened aggressiveness and metastatic potential of hypoxic tumor cells.

### 3.1. Angiogenesis

Growing tumors induce vascularization in tumor tissue since they need nourishment and oxygen to survive. Key regulators of this process are HIFs, which modulate the expression of genes such as VEGF-A (Vascular endothelial growth factor-A), SDF-1 (Stromal cell-derived factor-1), ANGPT2 (Angiopoietin-2), PGF (Placental growth factor), PDGF-B (Platelet-derived growth factor subunit B) and SCF (Stem cell factor), all crucial for neoangiogenesis [33]. However, these newly formed blood vessels often lack pericyte cover and smooth muscle tissue, which further exacerbates circulatory insufficiency [19]. Hypoxia induces a rapid and chaotic blood vessel formation during tumor progression [34] due to the imbalance of pro- and anti-angiogenic factors [35].

Hypoxia and HIF-1α also contribute to the bone marrow-derived cells’ recruitment and induce their differentiation into embryonic stem cells through VEGF regulation [36]. Moreover, they stimulate MMPs (matrix metalloproteinases) and contribute to blood vessel maturation through Ang-1, PDGF and TGF-β [34]. MMPs are involved in tumor invasion and metastasis formation, and to date, 28 human MMPs are known and have been categorized into subgroups based on their structure and substrate specificity. Collagenases (MMP-1, -8 and -13) are proteins associated with angiogenesis and their loss can lead to an irreversible matrix rupture [37].

Since a tumor requires neovascularization for growth, a large number of abnormal vessels provides nutrients to cancer cells. According to the literature, the abnormal tumor angiogenesis can contribute to the intravasation of cancer cells [38]. An earlier study has demonstrated that VEGF targeting therapy leads to the shrinkage of the primary tumor, but increases intratumoral hypoxia, resulting in a higher rate of detachment of the circulating tumor cell (CTC) cluster and metastasis formation. Interestingly, while HIF-1α is not essential for CTC cluster formation or metastasis, it still plays a role in this process [39].

### 3.2. Immune System Evasion

Tumor cells employ various strategies to evade attacks from the immune system through three main ways: (1) Immune effector cells fail to recognize tumor cells. (2) Tumor cells develop resistance and produce survival-inducing factors. (3) Tumor cells create an immunosuppressive microenvironment [40]. Tumor hypoxia can contribute to the immunosuppressive phenotype of both tumor cells and immune cells within the tumor [6]. The hypoxia-induced activation of HIFs contributes to tumor immune resistance, thereby hindering the cytotoxic T-cell-mediated lysis of cancer cells [41]. Alternatively, the increased expression of the cell surface molecule CD47 inhibits macrophage phagocytosis [42]. Among tumor-infiltrating lymphocytes, there are effector CD4^+^ and CD8^+^ T cells with strong anti-tumor properties as well as Treg cells, which play an important role in immune suppression. The hypoxic condition suppresses the non-antigen-specific proliferation of Treg cells in vitro, attributed to increased levels of iNOS and CD69, which downregulate T cell responses [43].

### 3.3. Invasion and Intravasation

During metastasis formation, tumor cells disseminate from the primary tumor and reach distant sites through blood-, lymphatic-, or cerebrospinal fluid circulation. The prerequisite for tumor cell migration is the epithelial–mesenchymal transition (EMT), in which epithelial cells lose their apico-basal polarization, intercellular adhesion and display mesenchymal features such as migration and invasion [44]. Invasion can occur in two forms: (1) single or (2) collective cell migration [45]. Cancer cells can switch from one form to another in response to environmental stimuli [46]. Among the collectively migrating cells, the leading cells are typically elongated at the front [47]. Hybrid epithelial–mesenchymal cells become leader cells during migration, characterized by a lack of cell polarity and partly a loss of complete or membrane-associated E-cadherin [48]. In many cell types, hypoxia or the overexpression of HIF is sufficient to induce EMT and invasion [49]. Cell–cell adhesions are responsible for the localization of cadherins at the intercellular junctions. The downregulation of E-cadherin allows cells to separate from neighboring cells, and thus increase the chance for migration. Reduced E-cadherin expression is observed in metastatic tumors, and in experimental animals, it has been shown to be sufficient to promote metastasis [50]. EMT is not always complete and during partial EMT, epithelial cells become motile, but do not completely lose cell–cell adhesion and their polarity [51]. Intermittent hypoxia increased the expression of HIF-1α and Nuclear factor erythroid 2-related factor 2 (NRF2), a key regulator of oxidative stress [52], which is involved in the stabilization of partial EMT in lung and bladder cancer cells [53]. EMT, which can be divided into three subtypes, plays a fundamental role in many physiological and pathological processes such as (1) in implantation, embryogenesis and tissue development; (2) in wound healing, tissue regeneration and fibrosis; and (3) in the metastasis formation of epithelial tumors [54]. The loss of functional E-cadherin, a characteristic of epithelial cells, and the appearance of mesenchymal markers, such as FSP1, α-smooth muscle actin, vimentin, desmin and N-cadherin [55], are accompanied by EMT, although the significance of each marker depends on the type of EMT. In murine primary tubular epithelial cells, exposure to hypoxia (1% O_2_) induces a more elongated cell morphology, while reducing ZO-1 (Zonula occludens-1) and elevating α-SMA (α-Smooth muscle actin) compared to normoxic cells [56]. HIF signaling in tumor cells triggers the expression of the CCL-5 (C-C motif ligand 5) chemokine and M-CSF1 (macrophage colony stimulating factor 1) cytokine, attracting macrophages and mesenchymal stem cells to the tumor microenvironment, thereby promoting cell invasion, migration and metastasis [57]. The extracellular matrix (ECM), a network of proteins and proteoglycans, serves various cellular functions and provides a scaffold for cells [58]. MMPs degrade the ECM and basal membrane, activating granulocytes and protecting inflammatory cells from apoptosis. Several genes like LOX, GYS1 (Glycogen synthase 1), PFKP (Phosphofructokinase, platelet), MXI1 (MAX interacting Protein 1), BNIP3L (BCL2 interacting protein 3 like), PGK1 (Phosphoglycerate kinase 1), RNASE4 (Ribonuclease 4), NDRG1 (N-Myc downstream regulated 1), SCL2A1 (solute carrier family 2 member 1), ANGPTL4 (Angiopoietin like 4) and LDHA (Lactate dehydrogenase A) are involved in the synthesis and degradation of ECM and are all regulated by HIF [59]. These genes promote the upregulation of vascular proangiogenic factors such as VEGFA and Ang1, as well as the proteolytic enzymes MT1-MMP and MMP1 [60].

The TGF-β (Transforming growth factor-β) signaling pathway is the most studied EMT pathway involving HIF-1α and SMAD (the main signal transducers of the receptors of TGF-β) as key players. In the case of the SMAD signaling cascade, the phosphorylation of TGFβRI activates SMAD signaling, leading to the transcription of EMT-associated genes following the oligomerization of SMAD2/3 with SMAD4 and their translocation to the nucleus [61]. Nowadays the attention is increasingly focused on the EMT transcription factors, like TWIST, Snail, Slug and Zeb1, which are regulated by HIF-1α. HIF-1α directly binds to the hypoxia response element (HRE) of Twist, upregulating its expression [62]. There are other EMT pathways, such as Wnt/β-catenin, which can increase hypoxia-induced EMT by HIF-1α signaling in hepatocellular carcinoma [63] and the hedgehog pathway, in which HIF-1α mediates EMT and invasion in pancreatic cells [64]. Hypoxia can induce EMT without HIF-1α activation through AMPK, PI3K-Akt-mTOR, MAPKs, NFκB or Notch pathway activation [61]. The connection between hypoxia and collective cell migration is unclear in cancer.

During intravasation, tumor cells enter the blood circulation. Hypoxia promotes VEGF-A production, inducing endothelial cell retraction to facilitate the entry of tumor cells into blood vessels. Recent studies suggest that hypoxia and ECM play an important role in altering the cell metabolism and tumor metastasis [65]. This process enhances intravasation, which is also stimulated by the SDF-1 chemokine and its receptor, CXCR4 (CXC-motif chemokine receptor 4) signaling, in a hypoxic environment [66].

### 3.4. Extravasation

During extravasation, tumor cells exit from the terminal capillaries of the blood and proliferate at a secondary site. The theory of Steven Paget about the “seed and soil” hypothesis suggests that the interaction between the cancer cells and a receptive microenvironment guides the metastatic spread of primary tumors to distant organs [67]. According to James Ewing, cancer cells are directed to these sites (which are the first organs in circulation from the primary site) by the lymphatic and circulatory systems [68]. It is now accepted that these theories are not controversial. Tumor hypoxia promotes the formation of clustered circulating tumor cells (CTCs) with a high metastatic ability, while proangiogenic therapy suppresses metastasis formation through the prevention of circulating tumor cell cluster generation [39]. We already know that a small proportion of CTCs (circulating tumor cells) are able to survive and induce metastasis, suggesting that the interaction between CTCs and the host blood microenvironment is essential for the development of CTC metastasis [69]. CTCs can exist singly or in a cluster form, and exhibit genotypic and phenotypic heterogeneity [70]. Cancer cells exposed to a low oxygen pressure in the primary tumor and upon entry into the bloodstream acquire a “hypoxic memory”, which is preserved even after the cells’ reoxygenation [71]. An earlier publication demonstrated that acute hypoxia correlates with transient increases in HIF-1α levels, elevated tumor numbers in the lung, transiently increased permeability in pulmonary microvessels and increased iNOS expression [72].

### 3.5. Colonization of Distant Tissue

The tumor cells colonize the new tissue or organ, where they begin to grow. Accumulating evidence suggest that stromal cells may play an important role in the formation of metastasis. The primary tumor can facilitate the formation of metastasis to distant organs and tissues through interactions with various stromal cells. This space, colonized by tumor cells, is referred to as the premetastatic niche [73]. The increased expression and secretion of lysyl-oxidase (LOX) by breast cancer cells results in the activation of HIF signaling. These proteins modify the collagen matrix of the lungs, thus recruiting bone marrow-derived cells, which, in turn, attract cancer cells to the lung tissue through chemokine production, thereby promoting metastasis [74]. In another study, it was found that hypoxia enhances the extravasation and homing of Waldenström macroglobulinemia cells to new bone marrow niches in vivo by increasing CXCR4/SDF-1-mediated chemotaxis and VLA4-mediated adhesion [75].

### 3.6. Growth of Metastasis

In the final step of metastasis, cancer cells begin to grow in colonized distant tissues and organs. In vivo studies on the liver metastasis of the hypoxia-tolerant tumor cell line L-CI.5s revealed the increased expression of HIF-1α and its signaling in syngeneic DBA/2 mice overexpressing TIMP-1 (Tissue inhibitor of matrix metalloproteinase-1), while the inhibition of HIF-1α led to a reduction in liver metastasis. In vitro experiments further demonstrated that TIMP-1 itself could induce HIF-1α and HIF-1-signaling [76]. These findings are correlated to ours, as the blocking of HIF-1α expression in a HT168-M1 melanoma cell line resulted in decreased metastasis formation in an animal model [5]

## 4. Therapy Resistance

Chemotherapy resistance attributed to hypoxia prevents anticancer drugs from acting on tumor cells efficiently, while radiotherapy and the production of reactive oxygen species (ROS) are ineffective under low oxygen conditions, resulting in radiotherapy resistance.

Hypoxia can contribute to drug resistance in different ways. (1) Most anticancer agents induce apoptosis, a process facilitated by free radicals, which are diminished in low oxygen conditions [77], decreasing the effect of these drugs. (2) Under hypoxia, ABC-transporter genes responsible for the development of resistance to anticancer drugs are induced [77]. (3) Moreover, hypoxia can modify metabolizing enzymes which are responsible for the activation or inactivation of anticancer drugs [78]. (4) The highly proliferating tumor cells induce new vessel formation under hypoxic conditions, which often results in a poorly organized vasculature. This inadequate vascularization impedes the perfusion and diffusion of anticancer drugs [79].

## 5. Anti-HIF Therapies

Although numerous compounds and drugs have been identified as a HIF-1α-blocking agent, a clinically approved selective inhibitor has yet to emerge, mostly due to issues with low selectivity, bad pharmacokinetics and high toxicity. To achieve the successful development of a selective, non-toxic HIF-1α inhibitor with better pharmacokinetic properties, it is necessary to use appropriate cancer models or employ combination therapy with already existing therapeutic agents in well-designed clinical trials.

## 6. Role of Small G Proteins and Cytoskeleton in Tumor Cell Metastasis under Hypoxia

The motility of tumor cells is essential during metastasis formation, in which adhesion points and cytoskeletal elements closely related to them are indispensable. Increasing evidence suggests that hypoxia causes cytoskeletal changes in different cell types [80]. The cytoskeleton is essential for eukaryotic cells as it regulates many cellular processes. One of the major components of the cytoskeleton is a highly conserved actin, which contributes to the cell shape, motility and polarity, cell division and the maintenance of multicellular tissue organization [81].

Small G proteins, approximately 21 kDa, are involved in a variety of cellular functions, including cell growth, migration, invasion, cytoskeleton rearrangement, cell survival and angiogenesis [82,83]. Small GTPases behave like enzymes and catalyze the hydrolysis of guanosine triphosphate (GTP) to guanosine diphosphate (GDP). The classic Rho GTPases, RhoA, Rac1 and cdc42, are a type of molecular switch with two conformational states, the inactive GDP-binding and the active GTP-binding forms. They can bind both GTP and GDP. Their function is regulated by guanine nucleotide exchange factors (GEFs), guanine nucleotide dissociation inhibitors (GDIs) and GTP-activated proteins (GAPs) [84]. GEFs promote the release of GDP from GTPase [85], and multiple GEFs can promote the GTP-GDP exchange by a GTPase [86]. GAPs enhance the activation of Rho GTPases to stimulate the hydroxylation of the bound GTP [85]. GDIs bind to Rho GTP in a GDP-bound form and inhibit their activity upon release into the cytosol. In hypoxia, Rho GTPases were overexpressed and localized in the membrane in a GTP-bound form, although they have no consensus HRE in their promoter regions. These findings were depended on ROS production, since the suppression of ROS activity abolished the ROS as well the upregulation of Rho proteins [87]. Rac1 accumulates in the nucleus, where it regulates the activity of transcription factors, and in the mitochondria, where it interacts with Bcl2 and triggers ROS production [88].

Vav1, belonging to GEFs, serves as an adaptor protein and a regulator of cytoskeleton organization as well. In lung cancer cells, Vav1 is continuously produced and degraded under normoxic conditions, while in a hypoxic condition, it is stabilized and required for HIF-1α accumulation [89]. ATP depletion due to a low oxygen level disrupts cell polarity and intercellular junctions by rearranging cytoskeletal actin [90].

**Figure 2:**
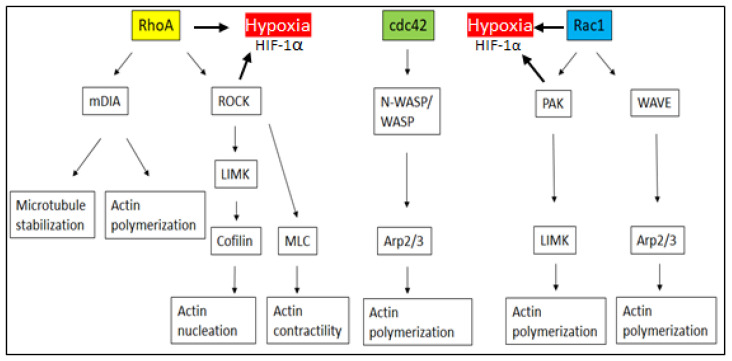
The relationship between small G-proteins, their downstream effectors and hypoxia-induced HIF-1α in hepatocellular carcinomas.

The Arp2/3 complex and Dia proteins are responsible for the nucleation of actin polymerization, with the Arp2/3 complex initiating filament formation and Dia protein elongating filaments. Arp2/3 activation by Rac1 and Cdc42 leads to lamellipodial actin network formation [91]. Cofilin involved in actin depolymerization is regulated by LIM kinases (LIMK), which are induced by P21-activated kinase (PAK) 1–4 and activated by Rac1 and Cdc42 or Rho kinase (ROCK) [92]. In hepatocellular carcinomas, it has been demonstrated that HIF-1α is not involved in the regulation of RhoA/ROCK and Rac1/PAK activity. However, the inhibition of RhoA and Rac1 reduces HIF-1α stabilization [93] (Figure 2). This suggests that the inhibition of Rac1 may be more relevant than the blocking of HIF-1α in modulating the metastatic potential of hypoxic tumors.

Increased Rac1 expression has been observed in breast, lung, colon, gastric, prostate, HCC and ovarian cancer [94,95,96,97,98]. Inhibiting Rac1 has demonstrated increased sensitivity to radiotherapy in pancreatic and breast tumors, highlighting its role in tumorigenesis, proliferation, metastasis and drug resistance [99]. Additionally, RhoA and Rac1 stabilizes the HIF-1α protein, suggesting the possible cross-talk between small G-proteins and HIF signaling pathways [93]. Nevertheless, although Rac1 plays an important role in the development of cancer, there is a lack of clinical studies associated with Rac1. However, different Rac1 inhibitors are available (Table 1).

An important effector of Rac1 is Wave2, a member of the WASP-family verprolin-homologous proteins, which regulate the actin cytoskeleton and play a role in cell migration and invasion. For instance, the ectopic overexpression of Rac1 in mouse melanoma cells has been shown to increase the invasiveness [106]. Another Rac1-regulated pathway in melanoma is PI3K-AKT, which also contributes to EMT [107] and migration [108,109]. Invadopodia are F-actin-rich protrusive structures formed by invasive cancer cells in contact with the extracellular matrix. They possess a proteolytic function and can focalize the secretion and accumulation of metalloproteinases [110]. The expression of wildtype Rac1 in cells promotes invadopodia formation, whereas a Rac1P29S mutation harboring cells, associated with a higher migration rate, exhibits suppressed invadopodia and matrix degradation, but enhanced lamellipodia formation [111]. In another study, melanoma cells expressing mutant Rac1 showed increased lamellipodium formation [112]. Additionally, Rac1 exerts its prometastatic effects by acting on the microtubule cytoskeleton. Constitutively active Rac1, via PAK1, interacts with microtubules and binds to the nucleoskeleton through the Linker of the Nucleoskeleton and Cytoskeleton (LINC) complex, promoting nuclear plasticity and the development of a more invasive phenotype [113].

Protein kinase C-z (PKC-z) mediates breast cancer cell invasion through Rac1 and RhoA pathways [114]. A high concentration of stromal cell-derived factor 1-α (SDF-1α) promotes the expression of Rac1 and mediates the migration and adhesion of breast cancer (BC) cells [115], and an oral contraceptive centchroman could inhibit the migration and invasion of breast cancer cells by blocking the Rac1-PAK1-b-catenin signal axis [116]. Rab23 is a member of the Ras-related small GTPase family, which can activate Rac1-TGFβ signal transduction and promotes EMT in hepatocellular carcinoma (HCC) cells [117]. POTEE, a member of the POTE anchor protein family E, promotes the invasion and migration of colorectal cancer and EMT by contributing the activation of Rac1 and Cdc42 [118]. Tualang honey preserved cellular adhesion (epithelial polarity) through the overexpression of β-catenin and E-cadherin and, in addition, inhibited the oral squamous cancer cell (OSCC) aggressiveness by downregulating TWIST1 and RAC1 [119]. Rac1 can influence the expression of EMT-related molecules and participate in the invasion and metastasis of CRC [96].

### Efficacy and Limitations of Rac1 Inhibition

The Rac1 inhibitor and its GEFs interaction is an important strategy to develop a clinical drug for inhibition. Targeting Rac1 reveals challenges. Rac1 inhibition has an essential role to block cell migration, invasion and the rearrangement of the actinskeleton of different tumor cells [120,121]. There are different methods to inhibit Rac1 activation: (1) blocking the GEF/Rac1 interaction, (2) reducing Rac1/GTP coupling, (3) hampering Rac1 localization and (4) targeting Rac1 effectors.

(1) NSC23766, the first selective RAC1–GEF blocking agent, inhibits the RAC1 interaction with TIAM1 and TRIO [122]. Regardless of its effects, NSC23766 potency is not sufficient to use it in clinical applications [123].
(2) EHT1864 blocks activation by directly binding to all Rac1 isoforms [124]. The inhibitor blocked the RAC1-mediated transformation [125], although showed untargeted effects as well [124].
(3) Regarding the importance of post-translational modifications mediating Rac1 subcellular localization and activation, several compounds which can block these lipid modifications have been developed. The geranylgeranyl transferases type I (GGTI) inhibitor demonstrated promising in vitro and preclinical results [126], exerting anti-tumorigenic effects in human pancreatic and non-small-cell lung cancer xenograft mouse models [127,128].
(4) The interaction between Rac1–effector was the most effective for blocking Rac1 without affecting other downstream signaling pathways [106]. The best described Rac1 effectors are the PAKs as they showed the sensibilization of the Rac1 P29S mutant melanoma cell lines and xenografts [129]. However, the clinical application is controversial using PAKs inhibitors. Targeting ARP2/3 or formins could be used in the treatment of Rac1 mutant tumors [112]. Selective PI3K inhibitors were able to prevent melanoma cell proliferation and migration driven by mutant Rac1 [130]. Overall, the therapeutical advantage of targeting Rac1–effector inhibition is proven, while more potent Rac1 inhibitors are still necessary.

The available Rac1 inhibitors have advantages and disadvantages. The NSC23766 was the first inhibitor that blocked the interaction between Rac1 and GEFs, but it did not interfere with the closely related targets Cdc42 or RhoA [122,131]. In HCC, NSC23766 can block the invasion and migration by inhibiting the CAMSAP2-dependent Rac1/JNK pathway, or the cysteine-rich domains-1 (LMCD1)-Rac1 pathway [132], while in NSCLC cells, it can regulate NF-kB activity, cell proliferation and cell migration [120]. The NSC23766 derivative EHop-016 has lower IC_50_. EHop-016 can reduce the Akt and Jun-kinase (JNK) activity and c-Myc and cyclin D expression, and increases the activity of caspase 3/7 in metastatic cancer cells, thereby affecting cell survival [133]. EHT1864 is a small molecule which interferes with the nucleotides binding to Rac1 and prevents the connections of GTPase to downstream effectors [134]. ZINC69391 is a specific Rac1 inhibitor, which interferes with the Rac1–GEF interaction by masking Trp56 residue on the Rac1 surface [135].

## 7. Conclusions

HIF-1α activation is strongly associated with invasion, metastasis and chemo- as well as radiotherapy resistance. Thereby, HIF-1 is recognized as a possible target for cancer therapy, and the beneficial effects of HIF-1 inhibitors have been confirmed in both preclinical and clinical studies. However, the efficacy of HIF-1 inhibitors has been inconsistent across clinical trials. Recently, attention has shifted to Rac1 and its inhibition as a strategy to impede tumor cell movement. Although the molecular link between hypoxia-induced HIF-1 alpha and small G-proteins remains unclear, the results of mainly in vitro studies so far suggest that Rac1 inhibition could be an effective complementary therapeutic target, emphasizing the need for potent Rac1 inhibitors.

## Figures and Tables

**Table 1:** Rac1 inhibitor molecules.

Rac1 Inhibitors	Description	References
NSC23766	The compound blocks activation by the guanine nucleotide exchange factors Trio and Tiam1, but does not affect interactions with RhoA or Cdc42. It blocks ADP-mediated platelet aggregation.	[100]
EHop-016	Derived from NSC23766. It has a lower IC_50_ than NSC23766.	[101]
Z62954982	Potent, selective and cell-permeable Rac1 inhibitor that is 4 times more effective than NSC23766. It disrupts the Rac1/Tiam1 complex and decreases cytoplasmic levels of active Rac1 (GTP-bound Rac1) without affecting the activity of other Rho GTPases (such as Cdc42 or RhoA).	[102]
ZINC69391	Specific Rac1 inhibitor. It acts by interfering with the interaction of Rac1 with Dock180, a relevant Rac1 activator in glioma invasion, and by reducing Rac1-GTP levels.	[103]
1A-116	Derived from ZINC69391. It is a Rac1 inhibitor, with antitumoral and antimetastatic effects in several types of cancer, such as breast cancer. It prevents Rac1-regulated processes involved in the primary tumorigenesis and metastatic processes.	[104]
EHT-1864	Inhibitor of Rac family GTPases. Blocks activation by direct binding to Rac1, Rac1b, Rac2 and Rac3. Inhibits Rac-, Ras- and Tiam-induced growth transformation of NIH-3T3 fibroblasts. Reduces β-amyloid peptide production in vivo.	[105]

Abbreviations: Tiam T-Lymphoma Invasion and metastasis-inducing protein, IC50 half maximal inhibitory concentration, NIH-3T3 mouse fibroblast.

## Abbreviations

Akt	Protein kinase B
AMPK	Adenosine monophosphate-activated protein kinase
Ang1	Angiopoietin-1
ANGPT2	Angiopoietin-2
ANGPTL4	Angiopoietin-like 4
α-SMA	alpha smooth muscle actin
ARNT	Aryl hydrocarbon receptor nuclear translocator (HIF-1β)
Arp2/3	Actin-related protein 2/3 complex
BNIP3L	BCL2 interacting protein 3 like
BC	Breast cancer
CCL-5	C-C motif ligand-5
Cdc42	Cell division control protein homolog
CH1	Cysteine/histidine-rich 1 domain
CML	Chronic myeloid leukemia
COP9	Constitutive photomorphogenesis mutant 9
COX2	Cyclooxygenase-2
CRC	Colorectal carcinoma
CSN5	COP9 signalosome subunit 5
C-TAD	C-terminal transactivation domain
CTC	Circulating tumor cell
CXCR4	CXC-motif chemokine receptor 4
Dia	Formin Diaphanous
ECM	Extracellular matrix
EGFR	Epidermal growth factor receptor
EMT	Epithelial–mesenchymal transition
FSP1	Ferroptosis suppressor protein 1
GAP	GTP-activated protein
GDI	Guanine nucleotide dissociation inhibitor
GDP	Guanosine–diphosphate
GEF	Guanine nucleotide exchange factor
GTP	Guanosine–triphosphate
GYS1	Glycogen synthase 1
HAF	Hypoxia-associated factor
HCC	Hepatocellular carcinoma
HER2	Human epidermal growth factor receptor 2
HIF	Hypoxia-inducible factor
HLH	Helix Loop Helix
HRE	Hypoxia-Response Element
LDHA	Lactate dehydrogenase A
LIMK	LIM domain kinase
LOX	Lysyl-oxidase
MAPK	Mitogen-activated protein kinase
MXI1	MAX-interacting Protein 1
MET	Mesenchymal–epithelial transition
MMP	Matrix metalloproteinase
MSCs	Mesenchymal stem cells
mTOR	Mammalian target of rapamycin
NDRG1	N-Myc downstream regulated 1
NF-κB	Nuclear factor k-light-chain-enhancer of activated B cells
LINC	Linker of nucleoskeleton and cytoskeleton NRF2—Nuclear factor erythroid 2-related factor 2
OCC	Oral squamous cancer cell
ODDD	Oxygen-dependent degradation domain
PAK	Serine/threonine protein kinase
PAS	Per ARNT Sim
PDGF-B	Platelet-derived growth factor subunit B
PGF	Placental growth factor
PGK1	Phosphoglycerate kinase 1
PI3K	Phosphoinositide 3 kinase
PFKP	Phosphofructokinase, platelet
Rac1	Ras-related C3 botulinum toxin substrate 1
RhoA	Ras homolog family member A
RNASE4	Ribonuclease 4
ROCK	Rho-associated protein kinase
ROS	Reactive oxygen species
SCF	Stem cell factor
SCL2A1	Solute carrier family 2 member 1
SDF-1	Stromal cell-derived factor 1
SMAD	Small worm phenotype (Caenorhabditis elegans) and MAD family (Mothers Against Decapentaplegic genes in Drosophila)
TADs	Transactivation domains
TIMP-1	Tissue inhibitor matrix metalloproteinase-1
TKI	Tyrosine kinase inhibitor
Treg	Regulatory T cell
VEGF	Vascular endothelial growth factor
VHL	von Hippel–Lindau
ZO-1	Zonula Occludens 1

## References

[1] Vajda J., Milojević M., Maver U., Vihar B. (2021). Microvascular Tissue Engineering-A Review. Biomedicines.

[2] Carreau A., El Hafny-Rahbi B., Matejuk A., Grillon C., Kieda C. (2011). Why Is the Partial Oxygen Pressure of Human Tissues a Crucial Parameter? Small Molecules and Hypoxia. J. Cell Mol. Med..

[3] Semenza G.L. (2012). Hypoxia-Inducible Factors in Physiology and Medicine. Cell.

[4] Tu J., Tu K., Xu H., Wang L., Yuan X., Qin X., Kong L., Chu Q., Zhang Z. (2020). Improving Tumor Hypoxia and Radiotherapy Resistance via in Situ Nitric Oxide Release Strategy. Eur. J. Pharm. Biopharm..

[5] Tátrai E., Bartal A., Gacs A., Paku S., Kenessey I., Garay T., Hegedűs B., Molnár E., Cserepes M.T., Hegedűs Z. (2017). Cell Type-Dependent HIF1 α-Mediated Effects of Hypoxia on Proliferation, Migration and Metastatic Potential of Human Tumor Cells. Oncotarget.

[6] Rankin E.B., Giaccia A.J. (2016). Hypoxic Control of Metastasis. Science.

[7] Hatfield S.M., Kjaergaard J., Lukashev D., Schreiber T.H., Belikoff B., Abbott R., Sethumadhavan S., Philbrook P., Ko K., Cannici R. (2015). Immunological Mechanisms of the Antitumor Effects of Supplemental Oxygenation. Sci. Transl. Med..

[8] Patel U., Pandey M., Kannan S., Samant T.A., Gera P., Mittal N., Rane S., Patil A., Noronha V., Joshi A. (2020). Prognostic and Predictive Significance of Nuclear HIF1α Expression in Locally Advanced HNSCC Patients Treated with Chemoradiation with or without Nimotuzumab. Br. J. Cancer.

[9] Irshad K., Mohapatra S.K., Srivastava C., Garg H., Mishra S., Dikshit B., Sarkar C., Gupta D., Chandra P.S., Chattopadhyay P. (2015). A Combined Gene Signature of Hypoxia and Notch Pathway in Human Glioblastoma and Its Prognostic Relevance. PLoS ONE.

[10] Cowman S.J., Fuja D.G., Liu X.-D., Tidwell R.S.S., Kandula N., Sirohi D., Agarwal A.M., Emerson L.L., Tripp S.R., Mohlman J.S. (2020). Macrophage HIF-1α Is an Independent Prognostic Indicator in Kidney Cancer. Clin. Cancer Res..

[11] Emami Nejad A., Najafgholian S., Rostami A., Sistani A., Shojaeifar S., Esparvarinha M., Nedaeinia R., Haghjooy Javanmard S., Taherian M., Ahmadlou M. (2021). The Role of Hypoxia in the Tumor Microenvironment and Development of Cancer Stem Cell: A Novel Approach to Developing Treatment. Cancer Cell Int..

[12] Almendros I., Gozal D. (2018). Intermittent Hypoxia and Cancer: Undesirable Bed Partners?. Respir. Physiol. Neurobiol..

[13] Rofstad E.K., Galappathi K., Mathiesen B., Ruud E.-B.M. (2007). Fluctuating and Diffusion-Limited Hypoxia in Hypoxia-Induced Metastasis. Clin. Cancer Res..

[14] Gaustad J.-V., Simonsen T.G., Roa A.M.A., Rofstad E.K. (2013). Tumors Exposed to Acute Cyclic Hypoxia Show Increased Vessel Density and Delayed Blood Supply. Microvasc. Res..

[15] Bhaskara V.K., Mohanam I., Rao J.S., Mohanam S. (2012). Intermittent Hypoxia Regulates Stem-like Characteristics and Differentiation of Neuroblastoma Cells. PLoS ONE.

[16] Miao Z.-F., Zhao T.-T., Wang Z.-N., Xu Y.-Y., Mao X.-Y., Wu J.-H., Liu X.-Y., Xu H., You Y., Xu H.-M. (2014). Influence of Different Hypoxia Models on Metastatic Potential of SGC-7901 Gastric Cancer Cells. Tumour Biol..

[17] Yoon D.W., So D., Min S., Kim J., Lee M., Khalmuratova R., Cho C.-H., Park J.-W., Shin H.-W. (2017). Accelerated Tumor Growth under Intermittent Hypoxia Is Associated with Hypoxia-Inducible Factor-1-Dependent Adaptive Responses to Hypoxia. Oncotarget.

[18] Saxena K., Jolly M.K. (2019). Acute vs. Chronic vs. Cyclic Hypoxia: Their Differential Dynamics, Molecular Mechanisms, and Effects on Tumor Progression. Biomolecules.

[19] Semenza G.L. (2012). Hypoxia-Inducible Factors: Mediators of Cancer Progression and Targets for Cancer Therapy. Trends Pharmacol. Sci..

[20] Tsai Y.-P., Wu K.-J. (2012). Hypoxia-Regulated Target Genes Implicated in Tumor Metastasis. J. Biomed. Sci..

[21] Choudhry H., Harris A.L. (2018). Advances in Hypoxia-Inducible Factor Biology. Cell Metab..

[22] He W., Batty-Stuart S., Lee J.E., Ohh M. (2021). HIF-1α Hydroxyprolines Modulate Oxygen-Dependent Protein Stability Via Single VHL Interface With Comparable Effect on Ubiquitination Rate. J. Mol. Biol..

[23] Lando D., Peet D.J., Gorman J.J., Whelan D.A., Whitelaw M.L., Bruick R.K. (2002). FIH-1 Is an Asparaginyl Hydroxylase Enzyme That Regulates the Transcriptional Activity of Hypoxia-Inducible Factor. Genes. Dev..

[24] Wolf D.A., Zhou C., Wee S. (2003). The COP9 Signalosome: An Assembly and Maintenance Platform for Cullin Ubiquitin Ligases?. Nat. Cell Biol..

[25] Koh M.Y., Powis G. (2009). HAF: The New Player in Oxygen-Independent HIF-1alpha Degradation. Cell Cycle.

[26] Xu R., Wang F., Yang H., Wang Z. (2022). Action Sites and Clinical Application of HIF-1α Inhibitors. Molecules.

[27] Albadari N., Deng S., Li W. (2019). The Transcriptional Factors HIF-1 and HIF-2 and Their Novel Inhibitors in Cancer Therapy. Expert. Opin. Drug Discov..

[28] Zhao J., Du F., Shen G., Zheng F., Xu B. (2015). The Role of Hypoxia-Inducible Factor-2 in Digestive System Cancers. Cell Death Dis..

[29] Fitzpatrick S.F. (2019). Immunometabolism and Sepsis: A Role for HIF?. Front. Mol. Biosci..

[30] Duan C. (2016). Hypoxia-Inducible Factor 3 Biology: Complexities and Emerging Themes. Am. J. Physiol. Cell Physiol..

[31] Gu Y.Z., Moran S.M., Hogenesch J.B., Wartman L., Bradfield C.A. (1998). Molecular Characterization and Chromosomal Localization of a Third Alpha-Class Hypoxia Inducible Factor Subunit, HIF3alpha. Gene Expr..

[32] Yamashita T., Ohneda O., Nagano M., Iemitsu M., Makino Y., Tanaka H., Miyauchi T., Goto K., Ohneda K., Fujii-Kuriyama Y. (2008). Abnormal Heart Development and Lung Remodeling in Mice Lacking the Hypoxia-Inducible Factor-Related Basic Helix-Loop-Helix PAS Protein NEPAS. Mol. Cell Biol..

[33] Lee S.H., Jeong D., Han Y.-S., Baek M.J. (2015). Pivotal Role of Vascular Endothelial Growth Factor Pathway in Tumor Angiogenesis. Ann. Surg. Treat. Res..

[34] Muz B., de la Puente P., Azab F., Azab A.K. (2015). The Role of Hypoxia in Cancer Progression, Angiogenesis, Metastasis, and Resistance to Therapy. Hypoxia (Auckl).

[35] de la Puente P., Muz B., Azab F., Azab A.K. (2013). Cell Trafficking of Endothelial Progenitor Cells in Tumor Progression. Clin. Cancer Res..

[36] Döme B., Hendrix M.J.C., Paku S., Tóvári J., Tímár J. (2007). Alternative Vascularization Mechanisms in Cancer: Pathology and Therapeutic Implications. Am. J. Pathol..

[37] Quintero-Fabián S., Arreola R., Becerril-Villanueva E., Torres-Romero J.C., Arana-Argáez V., Lara-Riegos J., Ramírez-Camacho M.A., Alvarez-Sánchez M.E. (2019). Role of Matrix Metalloproteinases in Angiogenesis and Cancer. Front. Oncol..

[38] Fukumura D., Kloepper J., Amoozgar Z., Duda D.G., Jain R.K. (2018). Enhancing Cancer Immunotherapy Using Antiangiogenics: Opportunities and Challenges. Nat. Rev. Clin. Oncol..

[39] Donato C., Kunz L., Castro-Giner F., Paasinen-Sohns A., Strittmatter K., Szczerba B.M., Scherrer R., Di Maggio N., Heusermann W., Biehlmaier O. (2020). Hypoxia Triggers the Intravasation of Clustered Circulating Tumor Cells. Cell Rep..

[40] Mittal D., Gubin M.M., Schreiber R.D., Smyth M.J. (2014). New Insights into Cancer Immunoediting and Its Three Component Phases--Elimination, Equilibrium and Escape. Curr. Opin. Immunol..

[41] Lee Y.-H., Bae H.C., Noh K.H., Song K.-H., Ye S., Mao C.-P., Lee K.-M., Wu T.-C., Kim T.W. (2015). Gain of HIF-1α under Normoxia in Cancer Mediates Immune Adaptation through the AKT/ERK and VEGFA Axes. Clin. Cancer Res..

[42] Zhang H., Lu H., Xiang L., Bullen J.W., Zhang C., Samanta D., Gilkes D.M., He J., Semenza G.L. (2015). HIF-1 Regulates CD47 Expression in Breast Cancer Cells to Promote Evasion of Phagocytosis and Maintenance of Cancer Stem Cells. Proc. Natl. Acad. Sci. USA.

[43] Kumar V., Gabrilovich D.I. (2014). Hypoxia-Inducible Factors in Regulation of Immune Responses in Tumour Microenvironment. Immunology.

[44] Nieto M.A., Huang R.Y.-J., Jackson R.A., Thiery J.P. (2016). EMT: 2016. Cell.

[45] Clark A.G., Vignjevic D.M. (2015). Modes of Cancer Cell Invasion and the Role of the Microenvironment. Curr. Opin. Cell Biol..

[46] Friedl P., Wolf K. (2003). Tumour-Cell Invasion and Migration: Diversity and Escape Mechanisms. Nat. Rev. Cancer.

[47] Chen B.-J., Wu J.-S., Tang Y.-J., Tang Y.-L., Liang X.-H. (2020). What Makes Leader Cells Arise: Intrinsic Properties and Support from Neighboring Cells. J. Cell Physiol..

[48] Bronsert P., Enderle-Ammour K., Bader M., Timme S., Kuehs M., Csanadi A., Kayser G., Kohler I., Bausch D., Hoeppner J. (2014). Cancer Cell Invasion and EMT Marker Expression: A Three-Dimensional Study of the Human Cancer-Host Interface. J. Pathol..

[49] Liu Y., Xu Y., Ji W., Li X., Sun B., Gao Q., Su C. (2014). Anti-Tumor Activities of Matrine and Oxymatrine: Literature Review. Tumour Biol..

[50] Serganova I., Mayer-Kukuck P., Huang R., Blasberg R. (2008). Molecular Imaging: Reporter Gene Imaging. Handb. Exp. Pharmacol..

[51] Yang J., Antin P., Berx G., Blanpain C., Brabletz T., Bronner M., Campbell K., Cano A., Casanova J., Christofori G. (2020). Guidelines and Definitions for Research on Epithelial-Mesenchymal Transition. Nat. Rev. Mol. Cell Biol..

[52] Malec V., Gottschald O.R., Li S., Rose F., Seeger W., Hänze J. (2010). HIF-1 Alpha Signaling Is Augmented during Intermittent Hypoxia by Induction of the Nrf2 Pathway in NOX1-Expressing Adenocarcinoma A549 Cells. Free Radic. Biol. Med..

[53] Bocci F., Tripathi S.C., Vilchez Mercedes S.A., George J.T., Casabar J.P., Wong P.K., Hanash S.M., Levine H., Onuchic J.N., Jolly M.K. (2019). NRF2 Activates a Partial Epithelial-Mesenchymal Transition and Is Maximally Present in a Hybrid Epithelial/Mesenchymal Phenotype. Integr. Biol. (Camb).

[54] Kalluri R., Weinberg R.A. (2009). The Basics of Epithelial-Mesenchymal Transition. J. Clin. Investig..

[55] Yang J., Weinberg R.A. (2008). Epithelial-Mesenchymal Transition: At the Crossroads of Development and Tumor Metastasis. Dev. Cell.

[56] Higgins D.F., Kimura K., Bernhardt W.M., Shrimanker N., Akai Y., Hohenstein B., Saito Y., Johnson R.S., Kretzler M., Cohen C.D. (2007). Hypoxia Promotes Fibrogenesis in Vivo via HIF-1 Stimulation of Epithelial-to-Mesenchymal Transition. J. Clin. Investig..

[57] Chaturvedi P., Gilkes D.M., Takano N., Semenza G.L. (2014). Hypoxia-Inducible Factor-Dependent Signaling between Triple-Negative Breast Cancer Cells and Mesenchymal Stem Cells Promotes Macrophage Recruitment. Proc. Natl. Acad. Sci. USA.

[58] Karamanos N.K., Theocharis A.D., Piperigkou Z., Manou D., Passi A., Skandalis S.S., Vynios D.H., Orian-Rousseau V., Ricard-Blum S., Schmelzer C.E.H. (2021). A Guide to the Composition and Functions of the Extracellular Matrix. FEBS J..

[59] Erler J.T., Bennewith K.L., Nicolau M., Dornhöfer N., Kong C., Le Q.-T., Chi J.-T.A., Jeffrey S.S., Giaccia A.J. (2006). Lysyl Oxidase Is Essential for Hypoxia-Induced Metastasis. Nature.

[60] Hielscher A., Qiu C., Porterfield J., Smith Q., Gerecht S. (2013). Hypoxia Affects the Structure of Breast Cancer Cell-Derived Matrix to Support Angiogenic Responses of Endothelial Cells. J. Carcinog. Mutagen..

[61] Tam S.Y., Wu V.W.C., Law H.K.W. (2020). Hypoxia-Induced Epithelial-Mesenchymal Transition in Cancers: HIF-1α and Beyond. Front. Oncol..

[62] Vaquero J., Guedj N., Clapéron A., Nguyen Ho-Bouldoires T.H., Paradis V., Fouassier L. (2017). Epithelial-Mesenchymal Transition in Cholangiocarcinoma: From Clinical Evidence to Regulatory Networks. J. Hepatol..

[63] Zhang Q., Bai X., Chen W., Ma T., Hu Q., Liang C., Xie S., Chen C., Hu L., Xu S. (2013). Wnt/β-Catenin Signaling Enhances Hypoxia-Induced Epithelial-Mesenchymal Transition in Hepatocellular Carcinoma via Crosstalk with Hif-1α Signaling. Carcinogenesis.

[64] Lei J., Ma J., Ma Q., Li X., Liu H., Xu Q., Duan W., Sun Q., Xu J., Wu Z. (2013). Hedgehog Signaling Regulates Hypoxia Induced Epithelial to Mesenchymal Transition and Invasion in Pancreatic Cancer Cells via a Ligand-Independent Manner. Mol. Cancer.

[65] Dekker Y., Le Dévédec S.E., Danen E.H.J., Liu Q. (2022). Crosstalk between Hypoxia and Extracellular Matrix in the Tumor Microenvironment in Breast Cancer. Genes.

[66] Jin F., Brockmeier U., Otterbach F., Metzen E. (2012). New Insight into the SDF-1/CXCR4 Axis in a Breast Carcinoma Model: Hypoxia-Induced Endothelial SDF-1 and Tumor Cell CXCR4 Are Required for Tumor Cell Intravasation. Mol. Cancer Res..

[67] Wu J., Long Y., Li M., He Q. (2021). Emerging Nanomedicine-Based Therapeutics for Hematogenous Metastatic Cascade Inhibition: Interfering with the Crosstalk between “Seed and Soil”. Acta Pharm. Sin. B.

[68] Pienta K.J., Robertson B.A., Coffey D.S., Taichman R.S. (2013). The Cancer Diaspora: Metastasis beyond the Seed and Soil Hypothesis. Clin. Cancer Res..

[69] Lin D., Shen L., Luo M., Zhang K., Li J., Yang Q., Zhu F., Zhou D., Zheng S., Chen Y. (2021). Circulating Tumor Cells: Biology and Clinical Significance. Signal Transduct. Target. Ther..

[70] Micalizzi D.S., Haber D.A., Maheswaran S. (2017). Cancer Metastasis through the Prism of Epithelial-to-Mesenchymal Transition in Circulating Tumor Cells. Mol. Oncol..

[71] Godet I., Shin Y.J., Ju J.A., Ye I.C., Wang G., Gilkes D.M. (2019). Fate-Mapping Post-Hypoxic Tumor Cells Reveals a ROS-Resistant Phenotype That Promotes Metastasis. Nat. Commun..

[72] Reiterer M., Colaço R., Emrouznejad P., Jensen A., Rundqvist H., Johnson R.S., Branco C. (2019). Acute and Chronic Hypoxia Differentially Predispose Lungs for Metastases. Sci. Rep..

[73] Kaplan R.N., Rafii S., Lyden D. (2006). Preparing the “Soil”: The Premetastatic Niche. Cancer Res..

[74] Erler J.T., Bennewith K.L., Cox T.R., Lang G., Bird D., Koong A., Le Q.-T., Giaccia A.J. (2009). Hypoxia-Induced Lysyl Oxidase Is a Critical Mediator of Bone Marrow Cell Recruitment to Form the Premetastatic Niche. Cancer Cell.

[75] Muz B., de la Puente P., Azab F., Ghobrial I.M., Azab A.K. (2015). Hypoxia Promotes Dissemination and Colonization in New Bone Marrow Niches in Waldenström Macroglobulinemia. Mol. Cancer Res..

[76] Muz B., de la Puente P., Azab F., Luderer M., Azab A.K. (2014). The Role of Hypoxia and Exploitation of the Hypoxic Environment in Hematologic Malignancies. Mol. Cancer Res..

[77] Jing X., Yang F., Shao C., Wei K., Xie M., Shen H., Shu Y. (2019). Role of Hypoxia in Cancer Therapy by Regulating the Tumor Microenvironment. Mol. Cancer.

[78] Legendre C., Hori T., Loyer P., Aninat C., Ishida S., Glaise D., Lucas-Clerc C., Boudjema K., Guguen-Guillouzo C., Corlu A. (2009). Drug-Metabolising Enzymes Are down-Regulated by Hypoxia in Differentiated Human Hepatoma HepaRG Cells: HIF-1alpha Involvement in CYP3A4 Repression. Eur. J. Cancer.

[79] Minchinton A.I., Tannock I.F. (2006). Drug Penetration in Solid Tumours. Nat. Rev. Cancer.

[80] Zieseniss A. (2014). Hypoxia and the Modulation of the Actin Cytoskeleton—Emerging Interrelations. Hypoxia (Auckl).

[81] Pollard T.D., Cooper J.A. (2009). Actin, a Central Player in Cell Shape and Movement. Science.

[82] Karlsson R., Pedersen E.D., Wang Z., Brakebusch C. (2009). Rho GTPase Function in Tumorigenesis. Biochim. Biophys. Acta.

[83] Maldonado M.D.M., Medina J.I., Velazquez L., Dharmawardhane S. (2020). Targeting Rac and Cdc42 GEFs in Metastatic Cancer. Front. Cell Dev. Biol..

[84] Stengel K.R., Zheng Y. (2012). Essential Role of Cdc42 in Ras-Induced Transformation Revealed by Gene Targeting. PLoS ONE.

[85] Bos J.L., Rehmann H., Wittinghofer A. (2007). GEFs and GAPs: Critical Elements in the Control of Small G Proteins. Cell.

[86] Iden S., Collard J.G. (2008). Crosstalk between Small GTPases and Polarity Proteins in Cell Polarization. Nat. Rev. Mol. Cell Biol..

[87] Turcotte S., Desrosiers R.R., Béliveau R. (2003). HIF-1alpha mRNA and Protein Upregulation Involves Rho GTPase Expression during Hypoxia in Renal Cell Carcinoma. J. Cell Sci..

[88] Kotelevets L., Chastre E. (2020). Rac1 Signaling: From Intestinal Homeostasis to Colorectal Cancer Metastasis. Cancers.

[89] Hong J., Min Y., Wuest T., Lin P.C. (2020). Vav1 Is Essential for HIF-1α Activation via a Lysosomal VEGFR1-Mediated Degradation Mechanism in Endothelial Cells. Cancers.

[90] Tam S.Y., Wu V.W.C., Law H.K.W. (2020). JNK Pathway Mediates Low Oxygen Level Induced Epithelial-Mesenchymal Transition and Stemness Maintenance in Colorectal Cancer Cells. Cancers.

[91] Ridley A.J. (2006). Rho GTPases and Actin Dynamics in Membrane Protrusions and Vesicle Trafficking. Trends Cell Biol..

[92] Maciver S.K., Hussey P.J. (2002). The ADF/Cofilin Family: Actin-Remodeling Proteins. Genome Biol..

[93] Zhang J.-G., Zhou H.-M., Zhang X., Mu W., Hu J.-N., Liu G.-L., Li Q. (2020). Hypoxic Induction of Vasculogenic Mimicry in Hepatocellular Carcinoma: Role of HIF-1 α, RhoA/ROCK and Rac1/PAK Signaling. BMC Cancer.

[94] Leng R., Liao G., Wang H., Kuang J., Tang L. (2015). Rac1 Expression in Epithelial Ovarian Cancer: Effect on Cell EMT and Clinical Outcome. Med. Oncol..

[95] Wang P., Liu G.-Z., Wang J.-F., Du Y.-Y. (2020). SNHG3 Silencing Suppresses the Malignant Development of Triple-Negative Breast Cancer Cells by Regulating miRNA-326/Integrin A5 Axis and Inactivating Vav2/Rac1 Signaling Pathway. Eur. Rev. Med. Pharmacol. Sci..

[96] Xia L., Lin J., Su J., Oyang L., Wang H., Tan S., Tang Y., Chen X., Liu W., Luo X. (2019). Diallyl Disulfide Inhibits Colon Cancer Metastasis by Suppressing Rac1-Mediated Epithelial-Mesenchymal Transition. Onco Targets Ther..

[97] Wu Y., Zhao Y., Huan L., Zhao J., Zhou Y., Xu L., Hu Z., Liu Y., Chen Z., Wang L. (2020). An LTR Retrotransposon-Derived Long Noncoding RNA lncMER52A Promotes Hepatocellular Carcinoma Progression by Binding P120-Catenin. Cancer Res..

[98] Venugopal S.V., Caggia S., Gambrell-Sanders D., Khan S.A. (2020). Differential Roles and Activation of Mammalian Target of Rapamycin Complexes 1 and 2 during Cell Migration in Prostate Cancer Cells. Prostate.

[99] Yan Y., Hein A.L., Etekpo A., Burchett K.M., Lin C., Enke C.A., Batra S.K., Cowan K.H., Ouellette M.M. (2014). Inhibition of RAC1 GTPase Sensitizes Pancreatic Cancer Cells to γ-Irradiation. Oncotarget.

[100] Levay M., Krobert K.A., Wittig K., Voigt N., Bermudez M., Wolber G., Dobrev D., Levy F.O., Wieland T. (2013). NSC23766, a Widely Used Inhibitor of Rac1 Activation, Additionally Acts as a Competitive Antagonist at Muscarinic Acetylcholine Receptors. J. Pharmacol. Exp. Ther..

[101] Montalvo-Ortiz B.L., Castillo-Pichardo L., Hernández E., Humphries-Bickley T., De la Mota-Peynado A., Cubano L.A., Vlaar C.P., Dharmawardhane S. (2012). Characterization of EHop-016, Novel Small Molecule Inhibitor of Rac GTPase. J. Biol. Chem..

[102] Yu M., Gong D., Lim M., Arutyunyan A., Groffen J., Heisterkamp N. (2012). Lack of Bcr and Abr Promotes Hypoxia-Induced Pulmonary Hypertension in Mice. PLoS ONE.

[103] Cardama G.A., Comin M.J., Hornos L., Gonzalez N., Defelipe L., Turjanski A.G., Alonso D.F., Gomez D.E., Menna P.L. (2014). Preclinical Development of Novel Rac1-GEF Signaling Inhibitors Using a Rational Design Approach in Highly Aggressive Breast Cancer Cell Lines. Anticancer. Agents Med. Chem..

[104] Trebucq L.L., Cardama G.A., Lorenzano Menna P., Golombek D.A., Chiesa J.J., Marpegan L. (2021). Timing of Novel Drug 1A-116 to Circadian Rhythms Improves Therapeutic Effects against Glioblastoma. Pharmaceutics.

[105] Hampsch R.A., Shee K., Bates D., Lewis L.D., Désiré L., Leblond B., Demidenko E., Stefan K., Huang Y.H., Miller T.W. (2017). Therapeutic Sensitivity to Rac GTPase Inhibition Requires Consequential Suppression of mTORC1, AKT, and MEK Signaling in Breast Cancer. Oncotarget.

[106] Colón-Bolea P., García-Gómez R., Casar B. (2021). RAC1 Activation as a Potential Therapeutic Option in Metastatic Cutaneous Melanoma. Biomolecules.

[107] Melendez J., Memtsa M., Stavroulis A., Fakokunde A., Yoong W. (2009). The Best Way to Determine the Best Way to Undertake a Hysterectomy. BJOG.

[108] Irie H.Y., Pearline R.V., Grueneberg D., Hsia M., Ravichandran P., Kothari N., Natesan S., Brugge J.S. (2005). Distinct Roles of Akt1 and Akt2 in Regulating Cell Migration and Epithelial-Mesenchymal Transition. J. Cell Biol..

[109] Wang J., Hirose H., Du G., Chong K., Kiyohara E., Witz I.P., Hoon D.S.B. (2017). P-REX1 Amplification Promotes Progression of Cutaneous Melanoma via the PAK1/P38/MMP-2 Pathway. Cancer Lett..

[110] Eddy R.J., Weidmann M.D., Sharma V.P., Condeelis J.S. (2017). Tumor Cell Invadopodia: Invasive Protrusions That Orchestrate Metastasis. Trends Cell Biol..

[111] Revach O.-Y., Winograd-Katz S.E., Samuels Y., Geiger B. (2016). The Involvement of Mutant Rac1 in the Formation of Invadopodia in Cultured Melanoma Cells. Exp. Cell Res..

[112] Mohan A.S., Dean K.M., Isogai T., Kasitinon S.Y., Murali V.S., Roudot P., Groisman A., Reed D.K., Welf E.S., Han S.J. (2019). Enhanced Dendritic Actin Network Formation in Extended Lamellipodia Drives Proliferation in Growth-Challenged Rac1(P29S) Melanoma Cells. Dev. Cell.

[113] Colón-Bolea P., García-Gómez R., Shackleton S., Crespo P., Bustelo X.R., Casar B. (2020). RAC1 Induces Nuclear Alterations through the LINC Complex to Enhance Melanoma Invasiveness. Mol. Biol. Cell.

[114] Smalley T., Islam S.M.A., Apostolatos C., Apostolatos A., Acevedo-Duncan M. (2019). Analysis of PKC-ζ Protein Levels in Normal and Malignant Breast Tissue Subtypes. Oncol. Lett..

[115] Pasquier J., Abu-Kaoud N., Abdesselem H., Madani A., Hoarau-Véchot J., Thawadi H.A., Vidal F., Couderc B., Favre G., Rafii A. (2015). SDF-1alpha Concentration Dependent Modulation of RhoA and Rac1 Modifies Breast Cancer and Stromal Cells Interaction. BMC Cancer.

[116] Khan S., Shukla S., Farhan M., Sinha S., Lakra A.D., Penta D., Kannan A., Meeran S.M. (2020). Centchroman Prevents Metastatic Colonization of Breast Cancer Cells and Disrupts Angiogenesis via Inhibition of RAC1/PAK1/β-Catenin Signaling Axis. Life Sci..

[117] Zhang L., Zhang B., You W., Li P., Kuang Y. (2020). Rab23 Promotes Hepatocellular Carcinoma Cell Migration Via Rac1/TGF-β Signaling. Pathol. Oncol. Res..

[118] Xu Q., Chen J., Peng M., Duan S., Hu Y., Guo D., Geng J., Zhou J. (2020). POTEE Promotes Colorectal Carcinoma Progression via Activating the Rac1/Cdc42 Pathway. Exp. Cell Res..

[119] Al-Koshab M., Alabsi A.M., Bakri M.M., Naicker M.S., Seyedan A. (2020). Chemopreventive Activity of Tualang Honey against Oral Squamous Cell Carcinoma-in Vivo. Oral. Surg. Oral. Med. Oral. Pathol. Oral. Radiol..

[120] Gastonguay A., Berg T., Hauser A.D., Schuld N., Lorimer E., Williams C.L. (2012). The Role of Rac1 in the Regulation of NF-κB Activity, Cell Proliferation, and Cell Migration in Non-Small Cell Lung Carcinoma. Cancer Biol. Ther..

[121] Karpel-Massler G., Westhoff M.-A., Zhou S., Nonnenmacher L., Dwucet A., Kast R.E., Bachem M.G., Wirtz C.R., Debatin K.-M., Halatsch M.-E. (2013). Combined Inhibition of HER1/EGFR and RAC1 Results in a Synergistic Antiproliferative Effect on Established and Primary Cultured Human Glioblastoma Cells. Mol. Cancer Ther..

[122] Gao Y., Dickerson J.B., Guo F., Zheng J., Zheng Y. (2004). Rational Design and Characterization of a Rac GTPase-Specific Small Molecule Inhibitor. Proc. Natl. Acad. Sci. USA.

[123] Marei H., Malliri A. (2017). Rac1 in Human Diseases: The Therapeutic Potential of Targeting Rac1 Signaling Regulatory Mechanisms. Small GTPases.

[124] Dütting S., Heidenreich J., Cherpokova D., Amin E., Zhang S.-C., Ahmadian M.R., Brakebusch C., Nieswandt B. (2015). Critical Off-Target Effects of the Widely Used Rac1 Inhibitors NSC23766 and EHT1864 in Mouse Platelets. J. Thromb. Haemost..

[125] Shutes A., Onesto C., Picard V., Leblond B., Schweighoffer F., Der C.J. (2007). Specificity and Mechanism of Action of EHT 1864, a Novel Small Molecule Inhibitor of Rac Family Small GTPases. J Biol Chem.

[126] Navarro-Lérida I., Sánchez-Perales S., Calvo M., Rentero C., Zheng Y., Enrich C., Del Pozo M.A. (2012). A Palmitoylation Switch Mechanism Regulates Rac1 Function and Membrane Organization. EMBO J..

[127] Zimonjic D.B., Chan L.N., Tripathi V., Lu J., Kwon O., Popescu N.C., Lowy D.R., Tamanoi F. (2013). In Vitro and in Vivo Effects of Geranylgeranyltransferase I Inhibitor P61A6 on Non-Small Cell Lung Cancer Cells. BMC Cancer.

[128] Lu J., Chan L., Fiji H.D.G., Dahl R., Kwon O., Tamanoi F. (2009). In Vivo Antitumor Effect of a Novel Inhibitor of Protein Geranylgeranyltransferase-I. Mol. Cancer Ther..

[129] Araiza-Olivera D., Feng Y., Semenova G., Prudnikova T.Y., Rhodes J., Chernoff J. (2018). Suppression of RAC1-Driven Malignant Melanoma by Group A PAK Inhibitors. Oncogene.

[130] Uribe-Alvarez C., Guerrero-Rodríguez S.L., Rhodes J., Cannon A., Chernoff J., Araiza-Olivera D. (2021). Targeting Effector Pathways in RAC1(P29S)-Driven Malignant Melanoma. Small GTPases.

[131] Pickering K.A., Gilroy K., Cassidy J.W., Fey S.K., Najumudeen A.K., Zeiger L.B., Vincent D.F., Gay D.M., Johansson J., Fordham R.P. (2021). A RAC-GEF Network Critical for Early Intestinal Tumourigenesis. Nat. Commun..

[132] Li D., Ding X., Xie M., Huang Z., Han P., Tian D., Xia L. (2020). CAMSAP2-Mediated Noncentrosomal Microtubule Acetylation Drives Hepatocellular Carcinoma Metastasis. Theranostics.

[133] Castillo-Pichardo L., Humphries-Bickley T., De La Parra C., Forestier-Roman I., Martinez-Ferrer M., Hernandez E., Vlaar C., Ferrer-Acosta Y., Washington A.V., Cubano L.A. (2014). The Rac Inhibitor EHop-016 Inhibits Mammary Tumor Growth and Metastasis in a Nude Mouse Model. Transl. Oncol..

[134] Onesto C., Shutes A., Picard V., Schweighoffer F., Der C.J. (2008). Characterization of EHT 1864, a Novel Small Molecule Inhibitor of Rac Family Small GTPases. Methods Enzymol.

[135] Liang J., Oyang L., Rao S., Han Y., Luo X., Yi P., Lin J., Xia L., Hu J., Tan S. (2021). Rac1, A Potential Target for Tumor Therapy. Front. Oncol..

